# The Biopotential of *Bellardia trixago* in Replacing Synthetic Compounds for Health‐Promoting Applications: Is It a Promising Candidate?

**DOI:** 10.1002/fsn3.70109

**Published:** 2025-04-14

**Authors:** Mehmet Veysi Cetiz, Sakina Yagi, Kouadio Ibrahime Sinan, Ismail Senkardes, Ismail Koyuncu, Ozgur Yuksekdag, Giovanni Caprioli, Agnese Santanatoglia, Gianni Sagratini, Enver Saka, Gulsah Ozturk, Bengusu Hacer Akgul, Gokhan Zengin

**Affiliations:** ^1^ Department of Medical Biochemistry Faculty of Medicine, Harran University Turkey; ^2^ Department of Botany Faculty of Science, University of Khartoum Khartoum Sudan; ^3^ Department of Biology Science Faculty, Selcuk University Konya Turkey; ^4^ Department of Pharmaceutical Botany Faculty of Pharmacy, Marmara University Istanbul Turkey; ^5^ CHemistry Interdisciplinary Project (CHip), School of Pharmacy, University of Camerino Camerino Italy

**Keywords:** antioxidant, *B. trixago*, cytotoxicity, enzyme inhibition, in silico method, phenolic profile

## Abstract

*Bellardia trixago* (L.) All. (family Orobanchaceae) is a facultative hemiparasitic plant used traditionally to cure many diseases. The present study was designed to evaluate the phenolic constituents, antioxidant, enzyme inhibitory, and cytotoxic properties of the aerial parts of *B. trixago* . Ethyl acetate (EtOAc), ethanol (EtOH), and 70% EtOH extracts were prepared by maceration, while the aqueous extract was prepared by infusion. Rutin, 4‐hydroxy benzoic acid, ferulic acid, *p*‐coumaric acid, hyperoside, delphinidin 3,5 diglucoside, kaempferol, and isoquercitrin were identified in most extracts with variable concentrations, but generally, the EtOH or 70% EtOH accumulated the highest contents. The 70% EtOH extract displayed the best antiradical (DPPH = 13.55 mg TE/g; ATBS = 53.78 mg TE/g) and ion‐reducing (CUPRAC = 50.09 mg TE/g; FRAP = 24.73 mg TE/g) properties. The aqueous extract recorded the highest chelating iron power (12.91 mg EDTAE/g) while EtOAc and EtOH exerted the highest total antioxidant activity (2.29 and 2.49 mmol TE/g, *p* > 0.05). The best cholinesterase (anti‐AChE = 2.91 mg GALAE/g; and anti‐BchE = 2.62 mg GALAE/g) and α‐glucosidase (1.19 mmol ACAE/g) inhibition activity was recorded from the EtOH extract, while that against the α‐amylase was obtained from the EtOAc extract (0.69 mmol ACAE/g). The plant was most cytotoxic toward the HT‐29 cell line, where the best effect was exerted by the 70% EtOH (IC_50_ 38.42 μg/mL) extract. Furthermore, network pharmacology analysis revealed critical gene targets and pathways associated with the bioactive compounds. Molecular docking studies revealed favorable binding modes and interaction patterns of major compounds with key colon cancer‐related proteins, which were further supported by molecular dynamics simulations. As the first investigation into the phenolic composition and pharmacological effects of *B. trixago* , this study highlights its potential as a promising source of bioactive compounds with therapeutic relevance for oxidative stress‐related conditions.

## Graphical abstract

It is worth mentioning that the first report evaluating the phenolic profile and pharmacological potential of *B. trixago* indicated that the plants could be a promising source of bioactive molecules with therapeutic potential against oxidative stress diseases.

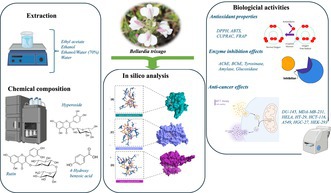

## 1 Introduction

The biological activity of plant extracts has recently been attracting attention. The main reason for interest is concern about the use of synthetic compounds (Soni et al. 2025). Among these biological activities, the enzyme‐inhibiting properties of plant extracts top the list. Inhibition of key enzymes by plant extracts may reduce the pathological signs of severe medical conditions. For example, in patients with diabetes, inhibition of amylase and glucosidase activity may control blood glucose levels. In addition, cholinesterase inhibition may control cognitive function in Alzheimer's disease. In this sense, some studies have been carried out to identify new sources of natural enzyme inhibitors instead of synthetic ones (Ahmed et al. 2025; Rauf and Jehan 2017).

Species belonging to the family Orobanchaceae are mainly parasites. They are annual or perennial herbs, rarely shrubs, and the family comprises about 2000 species distributed within 100 genera (Mutuku et al. 2021). Many of the Orobanchaceae species are used in traditional medicine and as wild food with significant nutritive values (Shi et al. 2020). However, despite the fact that many of the studied species were shown to be rich in phytoconstituents with unique chemical structures that can be useful subunits for organic synthesis, there is a lack of scientific exploration on the phytoconstituents and pharmacological properties of the majority of these parasitic herbs (Azab 2021; Luca et al. 2019; Scharenberg and Zidorn 2018; Shi et al. 2020).

*Bellardia trixago* (L.) All. (syn. *Bartsia trixago* L.) has been formerly classified in the family Scrophulariaceae and now it belongs to the family Orobanchaceae. *B. trixago* is a facultative hemiparasitic plant found mainly in the Mediterranean region but was also introduced in other regions like North and South America (Semiz and Günal 2023). Traditionally, it is used to treat HIV/AIDS, backache, menstrual problems, and as an antifebrile (Lamorde et al. 2010; Velasco‐Negueruela et al. 1995). Diterpenes were identified as the major constituent of the flower hexane extract (Barrero and Sånchez 1988). Epicuticular flavonoids were isolated from the whole plant, and their permethylated derivatives were shown to possess antifungal activity against *Cladosporium herbarum* (Tomas‐Barberan et al. 1990). From the aerial parts, iridoid glucosides were isolated, including the lignan glucoside dehydrodiconiferyl alcohol‐4‐0‐β‐D‐glucoside, aucubin, bartsioside, melampyroside, mussaenoside, and gardoside methyl ester (Ersöz et al. 1998; Soriano et al. 2022). Melampyroside was reported to be the most abundant iridoid in *B. trixago* and found to exert phytoxic activity against the obligate holoparasitic *Orobanche cumana* Wallr. (Soriano et al. 2022). Essential oil from dried aerial parts of a plant grown in Turkey contained cembrene (51.7%), phellandral (15.4%), and α‐ terpineol (14.5%) as the major constituents (Semiz and Günal 2023) while that from a species grown in Portugal contained (E–E)‐farnesyl acetone (42.1%), trixagol (8.0%), and 4‐vinyl guaiacol (5.4%) as the main constituents. Oil (mainly fatty acids) from the roots showed antifeeding activity against larvae of *Spodoptera littoralis* (Formisano et al. 2008). Recent advancements in bioinformatics and computational tools have facilitated a more comprehensive understanding of medicinal plants. Network pharmacology has emerged as a robust approach for identifying key interactions between bioactive compounds and disease‐related targets, facilitating a deeper exploration of therapeutic pathways (Duran et al. 2024; Kurt‐Celep et al. 2025; Zengin et al. 2025). In conjunction with molecular docking and molecular dynamics simulations, these methodologies yield insights into the binding affinities and stability of compound–protein complexes. Given the apparent lack of pharmacological potential in this plant, the current study was designed to identify the phenolic components in various aerial part extracts and evaluate their antioxidant activity through metal ion chelation, reduction, and free radical scavenging assays. Additionally, the cytotoxic effects of the extracts on a range of human carcinoma cell lines and normal cells were examined, along with their ability to inhibit enzymes associated with diabetes, skin hyperpigmentation, and Alzheimer's disease. Collectively, the results highlight the potential of natural sources of bioactive compounds as substitutes for synthetic alternatives.

## 2 Materials and Methods

### 2.1 Plant Samples

Botanical specimens were collected in 2023 from the Maltepe, Başıbüyük region of Istanbul, Turkey. Taxonomic identification was conducted by Dr. Ismail Senkardes, and a voucher specimen was preserved in the Marmara University Pharmacy Faculty herbarium (Voucher number: MARE‐23911). The aerial parts were shade‐dried at room temperature, pulverized, and stored in a light‐free environment.

### 2.2 Plant Extract Preparation

Four solvents—ethyl acetate, ethanol, a 70% ethanol/water mixture, and water—were employed in the extraction process. A 10‐g sample was macerated with 200 mL of the organic solvents for 24 h at room temperature. The aqueous extract was prepared by steeping 10 g of plant material in boiling water for 15 min. Organic extracts were concentrated under vacuum, whereas the aqueous extract was freeze‐dried.

### 2.3 Assay for Total Phenolic and Flavonoid Contents

Total phenolics (by Folin–Ciocalteu method) and flavonoids (by AlCl_3_ method) were quantified in our previous paper (Slinkard and Singleton 1977; Zengin et al. 2014). Gallic acid (GA) and rutin (R) served as reference standards in the experiments, with results reported as gallic acid equivalents (GAE) and rutin equivalents (RE).

### 2.4 HPLC‐ESI‐MS/MS Instrument and Analysis

The analysis of polyphenols was carried out by using high‐performance liquid chromatography coupled to tandem mass spectrometry (HPLC‐MS/MS) by using triple quadrupole as detector following a method previously developed (Mustafa et al. 2022; Santanatoglia, Caprioli, et al. 2023; Santanatoglia, Cespi, et al. 2023 Briefly, the analytical separation of the analytes was carried out on a Synergi Polar–RP C18 column preceded by a precolumn. The solvents used as mobile phases were water (A) and methanol, each with 0.1% formic acid at a flow rate of 0.8 mL/min in gradient elution mode.

### 2.5 Assays for In Vitro Antioxidant Capacity

As described in our previous paper (Grochowski et al. 2017), antioxidant tests were performed. DPPH, ABTS radical scavenging, CUPRAC, and FRAP results were milligrams of Trolox equivalents (TE) per gram. In millimoles of TE per gram of extract, the phosphomolybdenum (PBD) test measured antioxidant potential, and in EDTAE, metal chelating activity (MCA) was measured.

### 2.6 Inhibitory Effects Against Some Key Enzymes

Enzyme inhibition studies were performed on samples according to methods (Grochowski et al. 2017). Acarbose equivalents (ACAE) per gram of extract inhibited amylase and glucosidase, while milligrams of galantamine equivalents (GALAE) inhibited acetylcholinesterase (AChE) and butyrylcholinesterase. To evaluate tyrosinase inhibition per gram of extract, milligrams of kojic acid equivalents were used.

### 2.7 Cell Culture

The study employed cancer (DU‐145, MDA‐MB‐231, HELA, HT‐29, HCT‐116, A549, HGC‐27) and normal cell (HEK‐293) lines obtained from ATCC and stored in liquid nitrogen. The cells were cultured in DMEM‐F12/RPMI‐1640 media supplemented with 10% fetal bovine serum (FBS), 100 μg/mL streptomycin, and 100 IU/mL penicillin. The cells were maintained in incubators at 37°C under humidified conditions with 5% CO_2_.

### 2.8 Cell Viability Assay

The MTT assay was used to determine the cytotoxicity of the extracts. The cells were plated in a sterile 96‐well plate at 1 × 10^4^ cells per well and cultured for 24 h. The medium was then removed, and the extracts were applied at concentrations of 0–200 μg/mL for another 24 h. Next, 10 μL of MTT solution (0.5 mg/mL) was added to each well, followed by a 4‐h incubation. After removing the reagent, 100 μL of DMSO was added, and the absorbance was measured. The IC50 value was determined from the obtained data.

### 2.9 Disease Targets and Acquitting Intersection Targets

The plant composition was analyzed, and the compounds (gallic acid, prodelphinidin B, gallocatechin, prodelphinidin C, procyanidin B O‐hexoside, neochlorogenic acid, 3‐O‐quinic acid, catechin, epigallocatechin, chlorogenic acid, 3‐O‐feruloylquinic acid, caffeic acid, ampelopsin, epicatechin, 5‐O‐quinic acid, p‐coumaric acid, and prunin (naringenin‐7‐O‐glucoside)) were utilized to predict potential gene targets. The targets associated with these compounds were obtained from PubChem (https://pubchem.ncbi.nlm.nih.gov/) and The Comparative Toxicogenomics Database (CTD) (https://ctdbase.org/). Subsequently, all collected data files were consolidated, and duplicate entries were removed for a comprehensive screening. Furthermore, GeneCards (https://www.genecards.org/) and the CTD database were employed to predict genes associated with the identified pathways. The targets for diseases and compounds were then analyzed using the online tool Venny 2.1.0 (https://bioinfogp.cnb.csic.es/tools/venny/) to create a Venn diagram for identifying common genes. Subsequent to this, a more profound examination of these common genes was conducted to facilitate advanced analyses. (Cusumano et al. 2024; Zengin et al. 2024).

### 2.10 Protein–Protein Interaction Network Construction

In order to predict direct and indirect protein–protein interactions between target proteins, the STRING database (https://string‐db.org/) was utilized, with the human species “ *Homo sapiens* ” selected and a confidence score of 0.7 applied. The target interaction network diagram was consequently obtained and stored in TSV format. The data were then imported into Cytoscape 3.7.2 software for visualization. Finally, the network was examined using the “Network Analyzer” tool and CytoNCA plugin to determine the edges, nodes, betweenness, and degree value (connectivity), and the PPI network diagram was created. In addition, the CytoHubba plugin was used to identify the top 26 hub genes based on Maximal Clique Centrality (MCC) values of nodes and degrees for further study (Balkrishna et al. 2024).

### 2.11 Functional Annotation of Common Genes

Common targets were analyzed for functional annotation enrichment using DAVID (v6.8, https://david.ncifcrf.gov/home.jsp), covering biological processes (BP), cellular components (CC), molecular functions (MF), and KEGG pathway enrichment (Zengin et al. 2024). Potential regulatory pathways within the KEGG database were identified, with statistically significant KEGG pathways and GO terms determined using a *p*‐value threshold of 0.05 (Balkrishna et al. 2024). The top 20 KEGG pathways and top 10 GO terms were visualized in graphical plots based on count (Cetiz, Yagi, et al. 2024).

### 2.12 Molecular Docking

Molecular docking analysis was carried out to gain insights into the interaction of the major phytochemicals in the extract of *Bellardia trixago* . All 3D structures of selected proteins were downloaded from the protein databank (PDB) (https://www.rcsb.org/) as follows: Neurodegenerative disorders: AChE (PDB ID: 2Y2V) (Ahmed 2025), BChE (PDB ID: 3DJY) (Carletti et al. 2008); metabolic diseases: amylase (PDB ID: 2QV4) (Maurus et al. 2008), glucosidase (PDB ID: 3 W37) (Tagami et al. 2013); and skin‐related disorders: tyrosinase (PDB ID: 5M8O) (Lai et al. 2017) as well as colon adenocarcinoma‐associated proteins: PTGS2 (PDB ID: 5F19) (Lucido et al. 2016), IGFR1 (PDB ID: 3LW0) (Heinrich et al. 2010), EGFR (PDB ID: 1 M17) (Stamos et al. 2002), GTPase (KRas) (PDB ID: 4OBE) (Hunter et al. 2014), BRAF (PDB ID: 5VAM) (Nishiguchi et al. 2017), VEGFC (PDB ID: 2X1W) (Leppänen et al. 2010), and BIRC5 (PDB ID: 2QFA) (Jeyaprakash et al. 2007). The PlayMolecule web server (https://www.playmolecule.com/) was used to protonate each protein by computing the pKa of the titratable residues (Martínez‐Rosell et al. 2017). Moreover, all bond orders were corrected, and protein conformational energy minimization was done. Furthermore, all 3D structures of ligands were downloaded from the PubChem database (https://pubchem.ncbi.nlm.nih.gov/) (Pettersen et al. 2004) and optimized using UCSF Chimera (Pettersen et al. 2004). All hydrogen atoms were merged, and Gasteiger partial charges were added to all atoms to generate the pdbqt files. The docking grid files were generated either from the literature or using POCASA V1.1 (https://g6altair.sci.hokudai.ac.jp/g6/service/pocasa/) (Duran et al. 2024; Yu et al. 2010). AChE (PDB ID: 2y2v; X: 31.062, Y: 20.311, Z: 11.947; grid size: 22 Å × 30 Å × 40 Å) (Cetiz, Isah, et al. 2024), BChE (PDB ID: 3DJY; X: 44.794, Y: −19.63, Z: −25.227; grid size: 30 Å × 30 Å × 30 Å) (Yagi, Cetiz, et al. 2024), tyrosinase (PDB ID: 5M8O; X: −13.194, Y: 5.341, Z: −26.28; grid size: 26 Å × 26 Å × 28 Å) (Cusumano et al. 2024), amylase (PDB ID: 2QV4; X: 14.188, Y: 48.964, Z: 22.886; grid size: 28 Å × 28 Å × 24 Å) (Korpayev et al. 2024), glucosidase (PDB ID: 3 W37; X: 3.091, Y: −8.008, Z: −4.08; grid size: 42 Å × 52 Å × 54 Å) (Cetiz, Yagi, et al. 2024), PTGS2 (PDB ID: 5F19; X: −2.96, Y: −15.097, Z: 46.845; grid size: 40 Å × 54 Å × 76 Å), IGFR1 (PDB ID: 3LW0; X: −7.536, Y: −36.4, Z: −58.023; grid size: 102 Å × 96 Å × 72 Å), EGFR (PDB ID: 1 M17; X: 25.126, Y: 1.027, Z: 51.326; grid size: 64 Å × 34 Å × 28 Å), KRas (PDB ID: 4OBE; X: −2.96, Y: −15.097, Z: 46.845; grid size: 40 Å × 54 Å × 76 Å), BRAF (PDB ID: 5VAM; X: −24.533, Y: 43.972, Z: 43.972; grid size: 40 Å × 40 Å × 40 Å), VEGFC (PDB ID: 2X1W; X: −23.841, Y: 3.897, Z: 0.492; grid size: 124 Å × 126 Å × 114 Å), and BIRC5 (PDB ID: 2QFA; X: 47.377, Y: 8.176, Z: 32.712; grid size: 44 Å × 68 Å × 86 Å) (Yu et al. 2010). Furthermore, all ligand and protein preparations for docking were conducted using BIOVIA Discovery Studio and AutoDock V4.2.6 (Trott and Olson 2010). AutoDock Vina V1.1.2 (https://autodock.scripts.edu) (Morris et al. 2009) was used to search for ligand distinct conformations, with the exhaustiveness set to 32 (Trott and Olson 2010). In order to validate the accuracy of the docking process, the proteins were redocked with their cocrystallized ligands, and root mean square deviation (RMSD) values were calculated (Akpulat et al. 2025). To gain deeper insight into the protein/enzyme–ligand interactions, we employed the protein–ligand interaction profiler (PLIP) (https://plip‐tool.biotec.tu‐dresden.de/plip‐web/plip/index), which highlighted critical interactions, particularly hydrogen bonds (Angeles Flores et al. 2024; Llorent‐Martínez et al. 2025). These analysis methods confirmed that our docking results were characterized by precision and reliability.

### 2.13 Molecular Dynamic Simulation

Molecular dynamics simulations were executed using the CHARMM‐GUI platform (https://charmm‐gui.org). The system was prepared using the Solution Builder tool, in accordance with the guidelines established by Jo et al. (2008). The CHARMM36m force field was employed for protein parameterization, consistent with methods outlined by Yagi, Zengin et al. (2024) and Maier et al. (2015). TIP3P water molecules were used in a periodic boundary box, which ensured that the protein and the box edges were at least 10 Å apart. Counterions were added to bring the NaCl concentration down to 0–15 M and achieve electroneutrality. While the linear constraint solver (LINCS) algorithm was used to constrain bond lengths, the Verlet cutoff scheme was implemented to manage electrostatic and van der Waals interactions. To calculate the long‐range electrostatics, the particle mesh Ewald (PME) method was used (Angeles Flores et al. 2024). The energy minimization of the system was executed through the implementation of the steepest descent algorithm, thereby reducing potential energy fluctuations to below 1000 kJ/mol/nm (Korpayev et al. 2024). Equilibration was conducted under both the NVT and NPT conditions at 310 K to ensure thermodynamic stability. Subsequently, production simulations were run for a duration of 100 ns (ns) using GROMACS v2023.1 (Kurt‐Celep et al. 2024). These simulations focused on evaluating the stability, interactions, and therapeutic potential of the EGFR, KRas, and PTGS2 complexes with the delphinidin 3,5‐diglucoside ligand.

### 2.14 Statistical Analysis

Experiments were carried out in triplicate, and differences between extracts were analyzed using one‐way ANOVA (Tukey's test) in GraphPad Prism (v9.2). A *p*‐value < 0.05 was considered statistically significant. The biological activity dataset was scaled, centered, and subjected to PCA and clustered image maps (CIMs), with clustering performed using Ward's method and Euclidean distance. All multivariate analyses were conducted using R (v4.1.2).

### 2.15 Results and Discussion

#### 2.15.1 Total Phenolic (TPC) and Flavonoid (TFC) Contents

The TPC and TFC of the four extracts of *B. trixago* aerial parts were determined, and the results are presented in Table 1. The TPC was in the range of 12.00–17.54 mg GAE/g, with the highest content recorded in the 70% EtOH extract. It was apparent that flavonoids comprised the major content of all extracts, where the TFC ranged between 15.71 and 23.57 mg RE/g, and it was in the following order: 70% EtOH > EtOH > EtOAc > H_2_O.

**TABLE 1:** Total phenolic and flavonoid contents in extracts from *Bellardia trixago* aerial parts.

Extracts	TPC (mg GAE/g)	TFC (mg RE/g)
Ethyl acetate	13.50 ± 0.25^c^	18.76 ± 0.67^c^
Ethanol	12.00 ± 0.13^d^	22.86 ± 0.11^b^
70% Ethanol	17.54 ± 0.12^a^	23.57 ± 0.27^a^
Water	14.86 ± 0.11^b^	15.71 ± 0.19^d^

*Note:* Values are reported as mean ± SD of three parallel measurements. Different letters indicate significant differences among the tested extracts (*p* < 0.05).

Abbreviations: GA,Gallic acid equivalents; RE,Rutin equivalents.

Although it is expected that TPC is always higher than TFC, the nature of the extraction solvent and the procedure used for their determination affect the results as it was thought that flavonoids extracted by a solvent might not be totally determined in the TPC method (Yagi et al. 2023). Overall, the results showed that extracts were rich in flavonoids and 70% EtOH was the best solvent to recover TPC and TFC; however, these results from spectrophotometric analysis need to be confirmed using chromatographic techniques like HPLC or LC–MS/MS.

#### 2.15.2 Phenolic Profile

Analysis of the phenolic profile of the different extracts from *B. trixago* aerial parts was performed by LC/MS technique, and results are presented in Table 2. A total of 35 phenolic standards were used to detect their presence and concentrations in the four extracts. However, the majority of these compounds were not identified, and only 11, 10, 9, and 6 compounds were detected, respectively, in the EtOH, 70% EtOH, H_2_O, and EtOAc extracts, forming total concentrations of 873.23, 641.62, 385.86, and 250.20 mg/kg, respectively. The EtOH (328.40 mg/kg) and 70% EtOH (222.73 mg/kg) extracts were dominated by rutin, while 4‐hydroxy benzoic acid was a major compound in the aqueous (105.11 mg/kg) and EtOAc (104.45 mg/kg) extracts. The last compound was also found in high quantity in both the EtOH (106.68 mg/kg) and 70% EtOH (102.53 mg/kg) extracts. Additionally, these two extracts accumulated a high quantity of ferulic acid (EtOH = 110,81 mg/kg and 70% EtOH = 100.97 mg/kg). The four extracts also recorded a considerable amount of *p*‐coumaric acid (44.36–52.86 mg/kg) with the highest content detected in the EtOAc extract. Significant amounts of ferulic acid (95.7 mg/kg) and rutin (87.56 mg/kg) were found in the aqueous extract, while the EtOH extract was also characterized by the presence of hyperoside (101.98 mg/kg), delphinidin 3,5 diglucoside (64.36 mg/kg), and isoquercitrin (50.34 mg/kg). These compounds were also detected in the 70% EtOH extract but in lesser quantities. In fact, the EtOH and 70% EtOH extracts had the same chemical profile, except for chlorogenic acid and isorhamnetin, which were detected in the former, and a trace amount of kaempferol‐3‐glucoside was identified in the latter. However, it was clear that EtOH was the best solvent to recover a higher amount of the majority of the identified compounds.

**TABLE 2:** Chemical characterization of extracts from *Bellardia trixago* aerial parts. (Concentrations were expressed in mg/kg. n.d. means “not detected”).

COMPOUND	EtOAc	EtOH	70% EtOH	Water
Gallic acid	n.d.	6.79	6.42	n.d.
Neochlorogenic acid	n.d.	n.d.	n.d.	n.d.
Delphinidin 3‐galactoside	n.d.	n.d.	n.d.	n.d.
Catechin	n.d.	n.d.	n.d.	n.d.
Procyanidin B2	n.d.	n.d.	n.d.	n.d.
Chlorogenic acid	n.d.	2.84	n.d.	21.40
4‐Hydroxybenzoic acid	104.45	106.68	102.53	105.11
Epicatechin	n.d.	n.d.	n.d.	n.d.
Cyanidin‐3‐glucoside	n.d.	n.d.	n.d.	n.d.
Petunidin‐3‐glucoside	n.d.	n.d.	n.d.	n.d.
3‐Hydroxy benzoic acid	n.d.	n.d.	n.d.	n.d.
Caffeic acid	n.d.	n.d.	n.d.	n.d.
Vanillic acid	n.d.	n.d.	n.d.	n.d.
Pelargonidin‐3‐glucoside	n.d.	n.d.	n.d.	n.d.
Pelargonidin‐3‐rutinoside	n.d.	n.d.	n.d.	n.d.
Malvidin‐3‐galactoside	n.d.	n.d.	n.d.	n.d.
Syringic acid	n.d.	n.d.	n.d.	n.d.
Procyanidin A2	n.d.	n.d.	n.d.	n.d.
*p*‐Coumaric acid	52.86	49.38	44.36	45.16
Ferulic acid	25.91	110.81	100.97	95.78
Rutin	n.d.	328.40	222.73	87.56
Hyperoside	4.76	101.98	50.42	12.91
Isoquercitrin	n.d.	50.34	24.09	4.12
Delphinidin 3,5 Diglucoside	n.d.	64.36	36.33	12.53
Phloridzin	n.d.	n.d.	n.d.	n.d.
Quercitrin	n.d.	n.d.	n.d.	n.d.
Myricetin	n.d.	n.d.	n.d.	n.d.
Naringin	n.d.	n.d.	n.d.	n.d.
Kaempferol‐3‐glucoside	n.d.	n.d.	0.10	n.d.
Hesperidin	n.d.	n.d.	n.d.	n.d.
Ellagic acid	n.d.	n.d.	n.d.	n.d.
Quercetin	n.d.	n.d.	n.d.	n.d.
Phloretin	n.d.	n.d.	n.d.	n.d.
Kaempferol	52.14	45.69	53.66	1.30
Isorhamnetin	10.09	5.96	n.d.	n.d.
Total phenolic compounds	250.20	873.23	641.62	385.86

Additionally, it was shown that these compounds had substantial biological activities even though there were only a few identified compounds in the four extracts. For instance, rutin has anti‐inflammatory, antimicrobial, antidiabetic, hepatoprotective, nephroprotective, neuroprotective, antioxidant, antitumor, and testicular protective effects (Madkour et al. 2024). The phenolic acids, 4‐hydroxy benzoic acid, ferulic acid, and *p*‐coumaric acid, as well as the flavonoids kaempferol and isoquercitrin, are known for their therapeutic properties, such as antioxidant, anti‐inflammatory, antimicrobial, anticancer, and neurodegenerative properties (Alrumaihi et al. 2024; Gong and Zha 2023; Raj and Singh 2022; Valentová et al. 2014; Zaman et al. 2023). It has been demonstrated that hyperoside, a flavonol glycoside, can significantly help manage diabetes by scavenging free radicals, reducing damage to insulin secretion and glucose metabolism, preserving the structural integrity of the pancreas, and preventing β‐cell apoptosis (Y. Zhang et al. 2021). The anthocyanin delphinidin (detected in extracts in a glycosidic form) is found to exert antioxidant, anticancer, antiviral, anti‐inflammatory, and anti‐Alzheimer properties (Kowalczyk et al. 2024).

#### 2.15.3 Antioxidant Activity

The overproduction of reactive oxygen species (ROS) causes oxidative stress, which, in turn, causes a variety of illnesses, including cancer, diabetes, neurological disorders, and respiratory conditions. There is a constant need to investigate new natural sources of antioxidants because they counteract ROS (Forman and Zhang 2021). The antioxidant activity of the four extracts of *B. trixago* aerial parts was determined, and results are presented in Table 3. The 70% EtOH extract exerted significantly (*p* < 0.05) the highest antiradical (DPPH = 13.55 mg TE/g; ATBS = 53.78 mg TE/g) and ions reducing (CUPRAC = 50.09 mg TE/g; FRAP = 24.73 mg TE/g) properties. The aqueous extract recorded the second‐best values in the aforementioned assays and the highest chelating iron power (12.91 mg EDTAE/g). Although the other two extracts (EtOAc and EtOH) were less effective in scavenging radicals and reducing ions and did not chelate iron, they displayed the highest total antioxidant activity (2.29 and 2.49 mmol TE/g, *p* > 0.05). Overall, the 70% EtOH extract, followed by the aqueous one, recorded the best antiradical and ion reducing properties aligned with their TPC. Although the EtOH extract accumulated the highest concentrations of known antioxidant compounds like rutin, 4‐hydroxy benzoic acid, ferulic acid, *p*‐coumaric acid, kaempferol, and isoquercitrin, it exerted less antioxidant activity in all assays except in the PBD for determination of total antioxidant activity. This observation suggested the presence of other antioxidant compounds, not identified in the present study, in both the 70% EtOH and aqueous extracts, which may exert higher antioxidant properties than the identified compounds or may generate a synergistic effect and hence increase the antioxidant activity of the extracts. Previous studies revealed the richness of *B. trixago* with iridoid glycosides, and they were better recovered with hot water than EtOH (Suomi et al. 2000). Although iridoid glycosides were shown to be weakly correlated with the anti‐DPPH and Fe^+++^ reducing properties, they were suggested to complement the antioxidant activity of phenolic compounds (Kucharska et al. 2017). Furthermore, the presence of a considerable amount of chlorogenic acid, a potent antioxidant agent, in the aqueous extract may also significantly increase its antioxidant activity (Liang and Kitts 2015). On the other hand, the presence of compounds with antagonistic effects in the EtOH extract might also be responsible for its reduced antioxidant activity (Villanueva‐Bermejo et al. 2024). Overall, hydroethanol and aqueous extracts exerted the highest antioxidant property in most assays (5/6) while the less polar extracts (EtOAc and EtOH) displayed the best total antioxidant activity, and consequently, *B. trixago* aerial parts could be a promising source of antioxidant agents.

**TABLE 3:** Antioxidant properties of extracts from *Bellardia trixago* aerial parts.

Extracts	DPPH (mg TE/g)	ABTS (mg TE/g)	CUPRAC (mg TE/g)	FRAP (mg TE/g)	Chelating (mg EDTAE/g)	PBD (mmol TE/g)
Ethyl acetate	3.55 ± 0.74^c^	15.79 ± 1.49^d^	28.44 ± 2.59^c^	13.92 ± 0.42^c^	na	2.29 ± 0.01^a^
Ethanol	3.70 ± 0.15^c^	18.29 ± 0.11^c^	28.21 ± 0.42^d^	13.10 ± 0.04^c^	na	2.49 ± 0.17^a^
70% Ethanol	13.55 ± 0.30^a^	53.78 ± 0.65^a^	50.09 ± 0.95^a^	24.73 ± 0.69^a^	8.97 ± 0.85^b^	1.99 ± 0.18^b^
Water	11.24 ± 0.33^b^	48.49 ± 1.10^b^	36.24 ± 0.76^b^	18.86 ± 0.30^b^	12.91 ± 2.03^a^	1.69 ± 0.03^c^

*Note:* Values are reported as mean ± SD of three parallel measurements. Different letters indicate significant differences among the tested extracts (*p* < 0.05)

Abbreviations: EDTAE, EDTA equivalent; MCA, Metal chelating Activity; PBD, Phosphomolybdenum; TE, Trolox Equivalent.

#### 2.15.4 Enzyme Inhibition

Natural substances from plants with the capacity to inhibit enzymes have been proven to play a potential therapeutic role in the treatment of several diseases. For example, cholinesterase inhibitors like acetylcholinesterase (AChE) and butyrylcholinesterase (BChE) are effective for the treatment of Alzheimer's disease. Inhibition of α‐glucosidase and α‐amylase enzymes regulates blood sugar levels and hence offers an attractive strategy in the management of Type II diabetes. Tyrosinase (Tyr) inhibitors play an important role as depigmentation and antibrowning agents in cosmetics, medicinal food, and agriculture industries (Zolghadri et al. 2019). In the present study, the enzyme inhibitory activity of the four extracts of *B. trixago* aerial parts was determined, and results are presented in Table 4. EtOH extract revealed significantly (*p* < 0.05) the best anti‐AChE (2.91 mg GALAE/g) and anti‐BChE (2.62 mg GALAE/g) followed by the EtOAc extract (anti‐AChE = 2.52 mg GALAE/g; anti‐BChE = 1.76 mg GALAE/g). Furthermore, the three organic extracts exerted comparable anti‐Tyr activity (51.88–52.95 mg KAE/g, *p* > 0.05). Concerning the two enzymes associated with the breakdown of carbohydrates, the α‐amylase and α‐glucosidase enzymes were best inhibited by the EtOAc (0.69 mmol ACAE/g) and EtOH (1.19 mmol ACAE/g) extracts, respectively. The aqueous extract was either less effective or not active against the five tested enzymes. As mentioned before, the majority of the identified compounds were proven to possess interesting biological activities. Kaempferol and rutin were found to exert anti‐AChE (2.1 and 1.3 μM, respectively) and anti‐BChE (2.2 and 1.7 μM, respectively) activity (Szwajgier et al. 2020). The latter was also shown to inhibit the α‐amylase (53.66%) and α‐glucosidase (52.56%) enzymes (Dubey et al. 2017). 4‐hydroxy benzoic acid displayed significant anti‐AChE (78.60%; IC50 6.36 μmol/μmol AChE) (Budryn et al. 2022). Furthermore, the presence of *p*‐coumaric acid and ferulic acid in the four extracts might also partially contribute to their anti‐Tyr activity (Alifah et al. 2024; Li et al. 2020). Docking studies suggested that rutin can be a potent anti‐Tyr agent (Si et al. 2012). Ferulic acid was shown to effectively inhibit the α‐amylase (IC50 0.622 mg/mL) and α‐glucosidase (IC_50_ 0.866 mg/mL) enzymes (Zheng et al. 2020). Thus, *B. trixago* could be a promising source of bioactive molecules with enzyme inhibitory properties.

**TABLE 4:** Enzyme inhibitory properties of extracts from *Bellardia trixago* aerial parts.

Extracts	AChE (mg GALAE/g)	BChE (mg GALAE/g)	Tyrosinase (mg KAE/g)	Amylase (mmol ACAE/g)	Glucosidase (mmol ACAE/g)
Ethyl acetate	2.52 ± 0.02^b^	1.76 ± 0.11^b^	51.88 ± 3.21^a^	0.69 ± 0.02^a^	na
Ethanol	2.91 ± 0.02^a^	2.62 ± 0.10^a^	52.95 ± 1.36^a^	0.53 ± 0.05^b^	1.19 ± 0.11^a^
70% Ethanol	2.34 ± 0.05^c^	1.07 ± 0.26^c^	52.28 ± 1.46^a^	0.50 ± 0.02^b^	0.73 ± 0.03^b^
Water	1.01 ± 0.10^d^	0.40 ± 0.04^d^	14.97 ± 1.26^b^	0.09 ± 0.01^c^	na

*Note:* Values are reported as mean ± SD of three parallel measurements. Different letters indicate significant differences among the tested extracts (*p* < 0.05).

Abbreviations: ACAE, Acarbose equivalent; GALAE, Galantamine equivalent; KAE, Kojic acid equivalent; Na, not active.

#### 2.15.5 Cytotoxicity

Cancer is regarded as one of the primary causes of mortality globally. Due to the undesired side effect of synthetic chemotherapy, scientists are continually searching for alternative, effective, and safe anticancer agents. Plants contain a substantial reservoir of bioactive compounds that are crucial for the identification of new chemicals beneficial for cancer treatment (Welz et al. 2018). In the present study, the cytotoxic effect of the four extracts of *B. trixago* aerial parts was evaluated against the DU‐145 (prostate carcinoma), MDA‐MB‐231 (breast adenocarcinoma), HELA (cervix adenocarcinoma), HT‐29 (colon adenocarcinoma), HCT‐116 (colorectal carcinoma), A549 (lung adenocarcinoma), HGC‐27 (gastric carcinoma), and HEK‐293 (embryonic kidney epithelial) cells, and results are depicted in Table 5. The cytotoxic effect of the four extracts varied according to the cancer cells. However, the best toxic effect was observed, respectively, from the 70% EtOH (IC_50_ 38.42 μg/mL) and aqueous (IC_50_ 43.09 μg/mL) extracts against the HT‐29 cell line. The former extract also exerted the highest cytotoxic effect against HCT‐116 (IC_50_ 45.65 μg/mL) and A549 (IC_50_ 49.68 μg/mL) cells, while the latter displayed the best effect against the HGC‐27 cell. The EtOAc extract displayed the best effect against the DU‐145 (IC_50_ 44.35 μg/mL) followed by the aqueous extract (IC_50_ 47.48 μg/mL). The four extracts were less toxic to the MDA‐MB‐231 (IC_50_ 82.42–106.20 μg/mL) and HELA (IC_50_ 70.25–81.44 μg/mL) with the lowest IC_50_ values recorded from the aqueous and EtOH extracts, respectively. Furthermore, all extracts were less toxic to the normal HEK‐293 cell line (IC_50_ 77.17–107.84 μg/mL). It is worth mentioning that most of the identified compounds in the present study have been shown to suppress many cancer cells. For example, rutin was shown to effectively inhibit the HT‐29 cell (Al‐Ekaid et al. 2023). Ferulic acid suppressed the MDA‐MB‐231 cell in a concentration‐dependent manner (Zhang et al. 2016). Hyperoside suppressed the viability and induced apoptosis in the A549 in dose‐dependent manner (Fu et al. 2016). Also, delphinidin was reported to induce anticancer effect against the HCT‐116 (Zhang et al. 2021).

**TABLE 5:** Cytotoxic effects of *Bellardia trixago* extracts on cancer and normal cell lines (IC_50_ (μg/ml)).

CELLS	EA	EtOH	70% EtOH	Water
MDA‐MB‐231	106.20	85.29	102.00	82.42
HT‐29	80.13	76.23	38.42	43.09
HELA	70.29	70.25	72.00	81.44
DU‐145	44.35	50.60	64.86	47.48
HCT‐116	101.24	58.84	45.65	98.52
A549	96.54	79.68	49.68	74.14
HGC‐27	56.64	77.52	85.54	52.65
HEK‐293	85.65	107.84	77.17	89.97

#### 2.15.6 Exploratory Multivariate Analysis on Antioxidant and Enzyme Inhibitory Datasets

**FIGURE 1:**
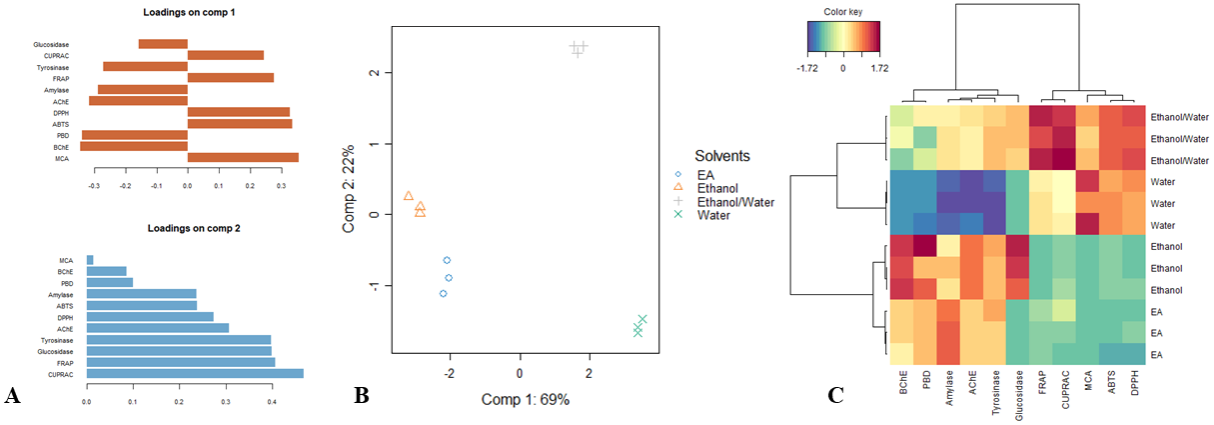
1‐Exploratory Principal component analysis. (A) Contribution of biological activities on the first two components of PCA. (B) Scatter plot showing the distribution of the samples in the factorial plan derived from the two retained components. (C) Clustered Image Map (Red color: High bioactivity. Blue color: low bioactivity) on biological activities dataset.

**FIGURE 2:**
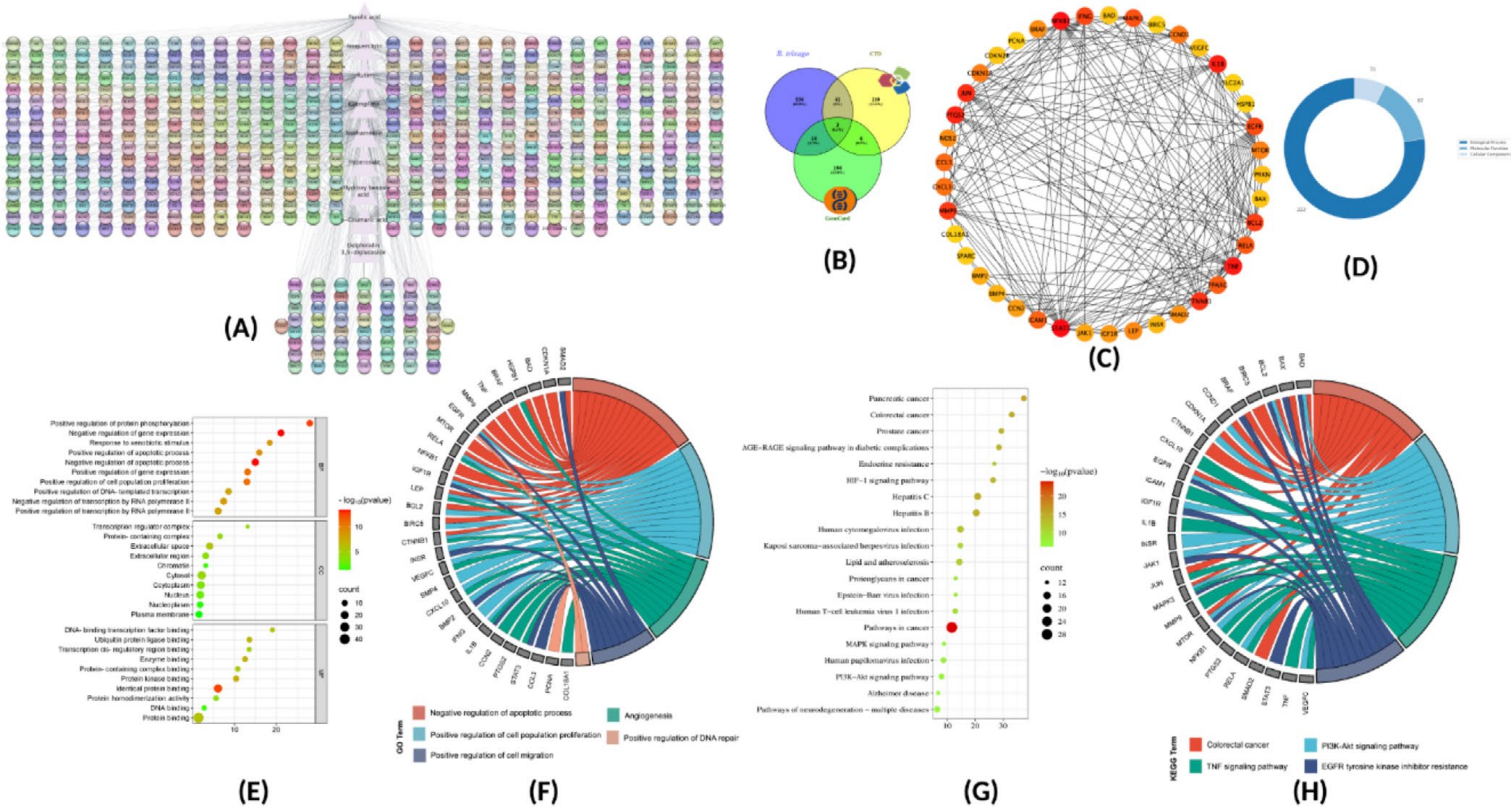
Network pharmacology workflow chart. (A) Gene‐target network of hub compounds from *B. trixago* . (B) Venn diagram showing overlapping genes related to colon adenocarcinoma and *B. trixago* . (C) MMC‐based hub targets with yellow‐to‐red gradient (low‐to‐high MMC scores). (D) Pie chart of all the GO terms. (E) Top 10 GO terms by enrichment. (F) Chord diagram of the top 5 GO terms. (G) Top 20 KEGG pathways with gene enrichment. (H) Chord diagram of the top 4 KEGG pathways and their gene targets.

The results of the principal component analysis (PCA) on the biological activities of Bellardia samples are presented in Figures 1 and 2. The eigenvalue screening indicated that the first two components sufficiently captured most of the data's variation, as suggested by Kaiser (1961). These components accounted for 69% and 22% of the variance, respectively.

The first component primarily reflected variations in six bioactivities. It was strongly and positively associated with MCA and both radical scavenging activities (ABTS and DPPH), while being negatively correlated with BChE, PBD, and AChE (Figure 2A). The second component differentiated the samples based on their CUPRAC, FRAP, glucosidase, and tyrosinase activities, with significant positive associations with these variables (Figure 2B).

An analysis of the scatter plots of Component 1 versus Component 2 (Figure 2D) revealed the emergence of three distinct groups. Afterward, the cluster image map, generated using the sample coordinates from the three dimensions of the PCA, confirmed the existence of the three groups (Figure 2D). Within the clusters, the samples in Cluster 3, which included the ethanolic and EA extracts, were distinguished by their highest BChE, PBD, Amylase, AChE, and tyrosinase activities. Clusters 1 (ethanol/water) exhibited the best radical scavenging activities (ABTS and DPPH) and reducing power (FRAP and CUPRAC), while Cluster 2 (water) recorded the strongest MCA activity.

#### 2.15.7 Disease Targets and Acquitting Intersection Targets

PubChem and CTD were used to predict potential gene targets for each hub phytocompound. In addition, after combining all data files and screening for duplication, 1159 distinct identities were discovered. Using Cytoscape, duplicate genes were removed, and the remaining genes were analyzed using the STRING plugin for direct gene mapping. This process yielded a total of 592 unique genes. The GeneCards database was used to predict genes associated with colorectal adenocarcinoma, yielding 297 target genes (Figure 2A). In addition, CTD was also used for the same purpose, and 160 targets were found, and GeneCard was also used for the same purpose, and 127 targets were found. The disease and drug targets were then used to create a Venn diagram using the online tool Venny 2.1.0 to identify common genes for further study. A total of 58 overlapping targets were found between differentially expressed genes (Figure 2B).

#### 2.15.8 Protein–Protein Interaction (PPI) Network Analysis

The projected 32 targets were imported into the STRING and Cytoscape software, respectively, for the PPI network development and analysis. Fifty‐eight nodes, or individual proteins, made up the protein–protein interaction (PPI) network complex. These nodes were connected by 214 edges, which showed the interactions or relationships between the proteins in the network. Higher degree nodes, or larger nodes, were thought to be more important to the network. The color of the nodes was determined based on their MCC (maximum clique centrality) scores; nodes in red represent highly significant and central targets within the network, while those in yellow indicate decreasing importance. The significance of the targets in the network was also assessed through degree scores, which represent the number of edges connected to a node. Among the 41 genes evaluated for their potential biological effects or functional roles (Figure 2C), the most prominent targets included TP53 (tumor protein 53), GSK3B (glycogen synthase kinase 3 beta), TNF (tumor necrosis factor), ESR1 (estrogen receptor 1), IL1B (interleukin 1 beta), BCL2 (B‐cell lymphoma 2), CASP3 (caspase 3), APP (amyloid beta precursor protein), HMOX1 (heme oxygenase 1), and PPARG (peroxisome proliferator‐activated receptor gamma). These genes occupy central roles in the network and are critical to various biological processes. A network was constructed to illustrate how *B. trixago* effectively targets the disease, demonstrating the relationships between compounds, targets, and pathways. Following a rigorous selection process, these specific genes were subjected to molecular docking investigations. These studies aim to explore their interactions, binding affinities, and potential therapeutic implications.

#### 2.15.9 GO Enrichment Analysis

The String tool produced 430 GO terms from the GO functional annotation investigation, including 31 cellular component (CC), 67 molecular function (MF), and 332 biological processes (BP) terms, all of which had a *p*‐value of 0.05. Each enrichment result's *p*‐value was calculated and varied from small to large. The percentage of enriched items in each section was shown in a pie chart, with BP making up the largest percentage (77.2%), followed by MF and CC at 15.6% and 7.2%, respectively (Figure 2D). This GO functional enrichment analysis revealed the most significant underlying therapeutic mechanisms within the context of biological processes (BP), cellular components (CC), and molecular functions (MF) of the GO system. Specifically, the biological processes (BP) observed included involvement in negative regulation of the apoptotic process, positive regulation of transcription by RNA polymerase II, negative regulation of gene expression, response to xenobiotics, and positive regulation of cell population proliferation. Within the category of cellular components (CC), the five most enriched terms were the transcriptional regulator complex, protein‐containing complex, extracellular region, chromatin, and cytosol. In the molecular functions (MF) category, the most enriched terms included protein binding, DNA binding, transcription factor binding, enzyme binding, and protein homodimerization (Figure 2E). The present study focuses on specific gene ontology (GO) terms. For instance, the study of colon adenocarcinoma is of particular interest, as it exemplifies the critical genes that are active in each pathway. BIRC5, EGFR, and BRAF represent genes that impede apoptotic processes, thereby promoting tumor cell survival and enhancing resistance to apoptosis. IGF1R, VEGFC, and EGFR have also been identified as playing a role in the positive regulation of the cell population proliferation pathway. The process of tumorosis is enhanced by an increase in mitogenic signaling as well as angiogenesis. Angiogenesis and new blood vessel formation are further facilitated by genes such as VEGFC and EGFR, which help to sustain the growth of tumors. The tumor‐stimulating effects are further augmented by the overactive response to xenobiotic stimuli, as indicated by the heightened expression of PTGS2. These genes also contribute to drug resistance and inflammation‐induced cancer (Figure 2F).

#### 2.15.10 KEGG Pathway Enrichment Analysis

**FIGURE 3:**
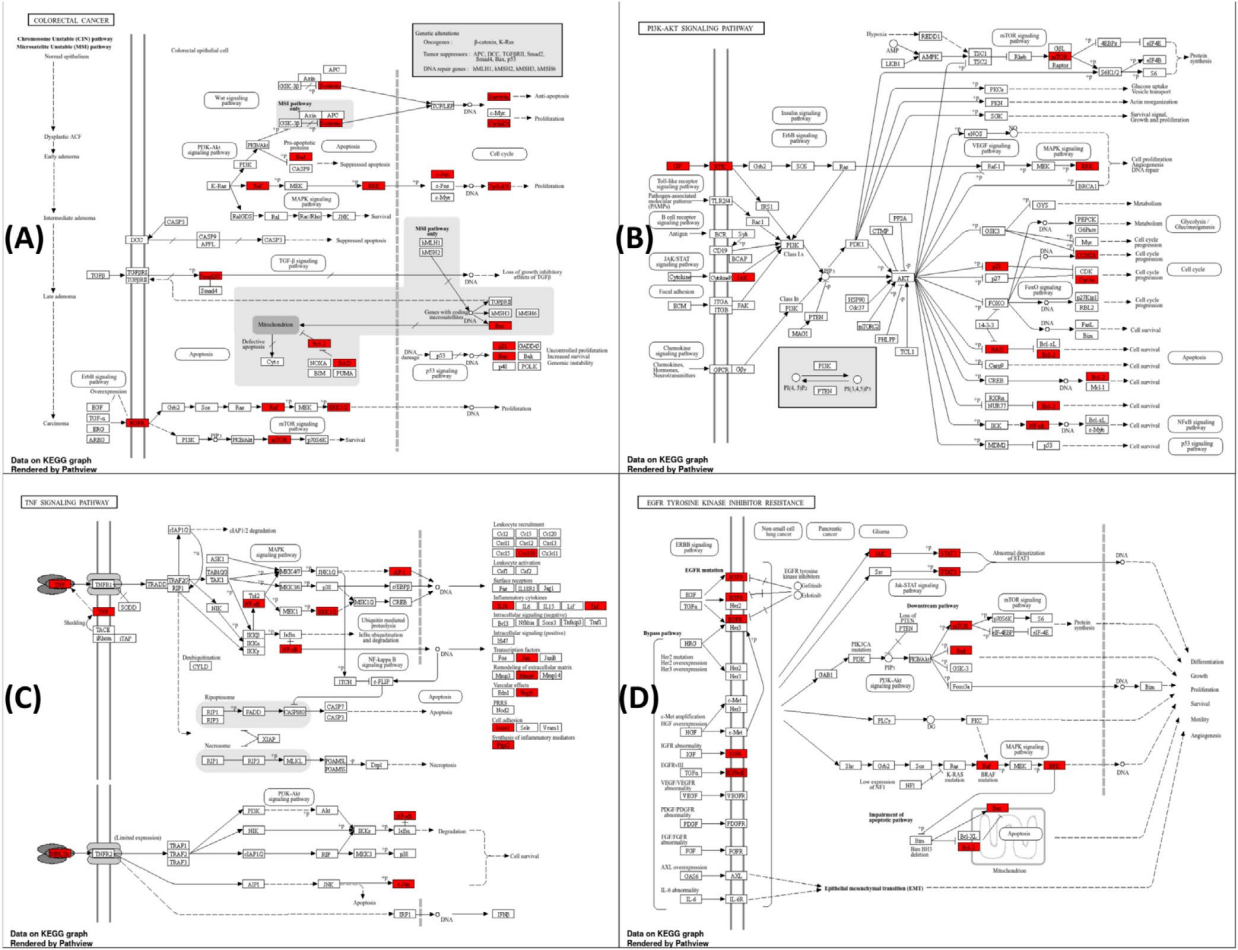
Functional pathways and key protein interrelationships (A) Colorectal cancer. (B) PI3K‐Akt signaling pathway (C) TNF signaling pathway. (D) EGFR tyrosine kinase inhibitor resistance.

A functional enrichment analysis was performed using the DAVID database for the 26 selected target genes, resulting in the identification of 132 pathways. Among these, the top 20 pathways were selected through KEGG enrichment analysis and ranked based on gene counts (Figure 2G). The most thoroughly delineated pathways encompassed “Pathways in cancer,” “Hepatitis C,” “Hepatitis B,” and “Human cytomegalovirus infection,”. Cancer‐specific pathways, including those associated with pancreatic cancer, colorectal cancer, and prostate cancer, were found to be highly enriched, thereby validating their significance in tumor emergence and dissemination. Noteworthy pathways include Kaposi sarcoma‐associated herpesvirus infection, human T‐cell leukemia virus 1 infection, and human papillomavirus infection. In this study, an investigation was conducted into four significant pathways: colorectal cancer (Figure 3A), PI3K‐Akt signaling pathway (Figure 3B), TNF signaling pathway (Figure 3C), and EGFR tyrosine kinase inhibitor resistance (Figure 3D). Particular note is the observation that three genes in MAPK3 are common to all four pathways. Furthermore, BRAF, BIRC5, CCND1, and CDKN1A are also important parts of colorectal cancer, and CCND1 and CDKN1A are also important parts of the PI3K‐Akt signaling pathway. IL1B, EGFR, MTOR, and MAPK3 play a crucial role in TNF signaling pathway. Additionally, STAT3 and IGF1R play a crucial role in the underlying mechanisms of resistance to the EGFR tyrosine kinase inhibitor resistance (Figures 2H and 3).

### 2.16 Molecular Docking Results

**FIGURE 4:**
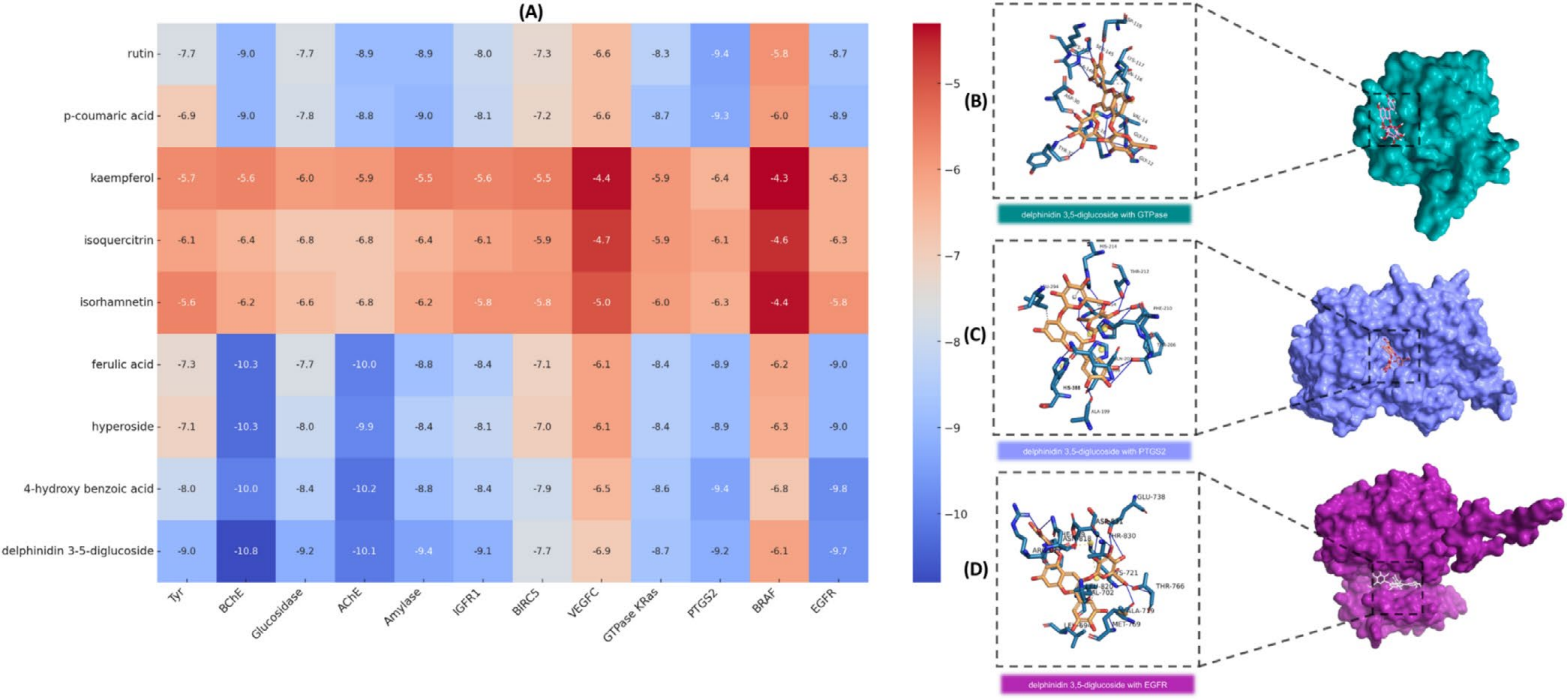
A comprehensive examination of the binding interactions between proteins and the compounds: (A) A visual representation of the docking scores for pertinent proteins and enzymes. (B) A molecular interaction analysis of delphinidin 3,5‐diglucoside with GTPase KRas. (C) A molecular interaction analysis of delphinidin 3,5‐diglucoside with PTGS2. (D) A molecular interaction analysis of delphinidin 3,5‐diglucoside with EGFR.

In this study, a comprehensive molecular docking approach was used to investigate the potential interactions between the bioactive compounds identified in *Bellardia trixago* and disease‐associated proteins, including those associated with neurodegenerative disorders, metabolic diseases, skin disorders, and colon adenocarcinoma. Chemical profiling identified several bioactive compounds, including kaempferol, hyperoside, isoquercitrin, delphinidin 3,5‐diglucoside, rutin, and isorhamnetin. These compounds were evaluated against key enzyme targets AChE, amylase, BChE, glucosidase, and tyrosinase—as well as cancer‐associated proteins, including EGFR, GTPase KRas, IGFR1, PTGS2, and VEGFC. Molecular docking analysis examined the binding affinities, free energy of association, RMSD values, and hydrogen bonding interactions of these phytochemicals with their respective targets. Figure 4 presents a comprehensive overview of all compounds with binding energies, while Table 6 focuses specifically on those compounds with binding energies equal to or lower than −8 kcal/mol. The computational results revealed a diverse range of binding energies, ranging from −10.8 to −4.3 kcal/mol, and RMSD values from 0.02 to 1.12 Å. These findings underscore the distinct affinity and conformational stability of each compound across various protein targets (Figure 4A, Table 6).

**TABLE 6:** The docking score (kcal/mol) and interacting residues of the enzyme and protein.

Compound	Target	PDB ID	Binding energy	RMSD	Interaction		Binding site
					Type	Number	
Delphinidin 3‐5‐diglucoside	Tyr	5m8o	−8.0	4.91	Hbond	11	LYS A:198;VAL A:211;TYR A:362;TYR A:362;TYR A:362;ARG A:374;ASN A:378;HIS A:381;GLY A:389;THR A:391;SER A:394
Rutin	Tyr	5m8o	−9.0	0.96	Hbond	12	HIS A:192;VAL A:211;GLU A:216;ARG A:321;TYR A:362;TYR A:362;TYR A:362;ARG A:374;ARG A:374;THR A:391;HIS A:392;HIS A:392
Kaempferol	BChE	3djy	−9.0	1.06	Hbond	5	ASP A:70;ASP A:70;GLU A:197;TYR A:332;HIS A:438
Isorhamnetin	BChE	3djy	−9.0	0.14	Hbond	3	ASP A:70;ASP A:70;TYR A:332
Hyperoside	BChE	3djy	−10.3	0.33	Hbond	7	TRP A:82;GLY A:115;TYR A:128;GLU A:197;ALA A:199;TYR A:332;HIS A:438
Isoquercitrin	BChE	3djy	−10.3	0.88	Hbond	11	GLY A:115;GLY A:115;GLY A:116;THR A:120;TYR A:128;ALA A:199;ALA A:199;LEU A:286;TYR A:332;TYR A:332;HIS A:438
Delphinidin 3‐5‐diglucoside	BChE	3djy	−10.0	0.51	Hbond	7	ASN A:68;ASP A:70;GLY A:115;GLU A:197;GLU A:197;THR A:284;HIS A:438
Rutin	BChE	3djy	−10.8	1.05	Hbond	11	ASP A:70;GLY A:78;GLY A:116;GLY A:116;THR A:120;THR A:120;TYR A:128;ALA A:199;ALA A:199;SER A:287;HIS A:438
Isoquercitrin	Glucosidase	3w37	−8.0	0.58	Hbond	7	ASN A:417;ASN A:417;GLY A:424;ASP A:440;ASP A:440;ASP A:443;ASN A:517
Delphinidin 3‐5‐diglucoside	Glucosidase	3w37	−8.4	12.99	Hbond	13	ASP A:232;ASP A:232;ASP A:232;ASP A:232;ASN A:237;PHE A:476;ASN A:496;SER A:497;SER A:505;SER A:505;LYS A:506;ASP A:568;ASP A:568
Rutin	Glucosidase	3w37	−9.2	0.69	Hbond	12	ASP A:232;ALA A:234;PHE A:476;ASN A:496;ASN A:496;SER A:497;SER A:497;SER A:505;SER A:505;LYS A:506;ARG A:552;ASP A:568
Kaempferol	AChE	2y2v	−8.9	0.53	Hbond	5	ASP A:74;GLY A:121;GLY A:122;TYR A:124;PHE A:295
Isorhamnetin	AChE	2y2v	−8.8	0.59	Hbond	2	TYR A:72;HIS A:447
Hyperoside	AChE	2y2v	−10.0	0.69	Hbond	14	ASP A:74;TRP A:86;GLY A:121;GLY A:122;TYR A:124;TYR A:124;TYR A:133;TYR A:133;GLU A:202;PHE A:295;ARG A:296;ARG A:296;TYR A:341;HIS A:447
Isoquercitrin	AChE	2y2v	−9.9	0.58	Hbond	11	TRP A:86;GLY A:121;GLY A:122;TYR A:124;TYR A:124;SER A:125;TYR A:133;PHE A:295;ARG A:296;TYR A:341;HIS A:447
Delphinidin 3‐5‐diglucoside	AChE	2y2v	−10.2	0.12	Hbond	16	TYR A:72;ASN A:87;GLY A:120;GLY A:120;GLY A:121;GLY A:122;TYR A:124;SER A:125;TYR A:133;GLU A:202;GLU A:202;PHE A:295;ARG A:296;ARG A:296;TYR A:341;HIS A:447
Rutin	AChE	2y2v	−10.1	0.02	Hbond	11	TYR A:72;ASP A:74;THR A:75;THR A:75;LEU A:76;TYR A:124;SER A:293;PHE A:295;ARG A:296;ARG A:296;TYR A:337
Kaempferol	Amylase	2qv4	−8.9	0.60	Hbond	5	GLN A:63;ARG A:195;ARG A:195;ASP A:197;HIS A:299
Isorhamnetin	Amylase	2qv4	−9.0	1.04	Hbond	6	GLN A:63;HIS A:101;ARG A:195;ARG A:195;HIS A:299;ASP A:300
Hyperoside	Amylase	2qv4	−8.8	0.05	Hbond	6	GLN A:63;HIS A:299;ASP A:300;ASP A:300;ASP A:300;HIS A:305
Isoquercitrin	Amylase	2qv4	−8.4	1.09	Hbond	7	GLN A:63;THR A:163;ARG A:195;ARG A:195;HIS A:201;HIS A:299;HIS A:305
Delphinidin 3‐5‐diglucoside	Amylase	2qv4	−8.8	0.98	Hbond	8	GLN A:63;GLN A:63;THR A:163;ARG A:195;LYS A:200;HIS A:201;HIS A:299;ASP A:300
Rutin	Amylase	2qv4	−9.4	0.97	Hbond	11	GLN A:63;ARG A:195;ASP A:197;ALA A:198;LYS A:200;ILE A:235;ILE A:235;HIS A:299;ASP A:300;HIS A:305;HIS A:305
Kaempferol	IGFR1	3lw0	−8.0	0.94	Hbond	3	LYS A:1033;VAL A:1063;ASP A:1135
Isorhamnetin	IGFR1	3lw0	−8.1	0.25	Hbond	3	VAL A:1063;HIS A:1133;ASP A:1135
Hyperoside	IGFR1	3lw0	−8.4	1.08	Hbond	6	ARG A:1134;ASP A:1135;ASN A:1140;ASN A:1140;ASP A:1153;MET A:1156
Isoquercitrin	IGFR1	3lw0	−8.1	7.31	Hbond	9	LYS A:1033;GLU A:1050;HIS A:1133;ASP A:1135;ASN A:1140;ASP A:1153;ASP A:1153;ASP A:1153;MET A:1156
Delphinidin 3‐5‐diglucoside	IGFR1	3lw0	−8.4	0.41	Hbond	9	SER A:1009;SER A:1009;ASP A:1135;ASN A:1140;ASN A:1140;ASP A:1153;MET A:1156;THR A:1157;THR A:1157
Rutin	IGFR1	3lw0	−9.1	0.98	Hbond	8	ASN A:1049;GLU A:1050;GLU A:1050;HIS A:1133;HIS A:1133;MET A:1156;THR A:1157;THR A:1157
Kaempferol	GTPase KRas	4obe	−8.3	0.78	Hbond	7	SER A:17;ASN A:116;ASN A:116;LYS A:117;SER A:145;ALA A:146;LYS A:147
Isorhamnetin	GTPase KRas	4obe	−8.7	0.80	Hbond	8	GLY A:13;SER A:17;SER A:17;ASN A:116;ASN A:116;LYS A:117;ALA A:146;LYS A:147
Hyperoside	GTPase KRas	4obe	−8.4	0.55	Hbond	11	GLY A:13;SER A:17;GLU A:31;TYR A:32;ASN A:116;ASN A:116;LYS A:117;LYS A:117;SER A:145;ALA A:146;LYS A:147
Isoquercitrin	GTPase KRas	4obe	−8.4	1.08	Hbond	10	GLY A:13;SER A:17;GLU A:31;TYR A:32;ASN A:116;ASN A:116;LYS A:117;LYS A:117;ALA A:146;LYS A:147
Delphinidin 3‐5‐diglucoside	GTPase KRas	4obe	−8.6	0.83	Hbond	15	GLY A:12;GLY A:13;VAL A:14;GLY A:15;LYS A:16;LYS A:16;ASP A:30;TYR A:32;TYR A:32;ASN A:116;LYS A:117;ASP A:119;SER A:145;ALA A:146;LYS A:147
Rutin	GTPase KRas	4obe	−8.7	1.12	Hbond	11	GLY A:12;SER A:17;ASP A:30;ASN A:86;ASN A:116;LYS A:117;LYS A:117;SER A:145;SER A:145;ALA A:146;LYS A:147
Kaempferol	PTGS2	5F19	−9.4	27.23	Hbond	3	CYS A:47;GLY A:135;GLU A:465
Isorhamnetin	PTGS2	5F19	−9.3	31.16	Hbond	3	ALA A:199;HIS A:386;TRP A:387
Hyperoside	PTGS2	5F19	−8.9	0.05	Hbond	6	ASN A:34;ASN A:34;CYS A:47;SER A:49;SER A:49;LYS A:137
Isoquercitrin	PTGS2	5F19	−8.9	30.43	Hbond	7	ASN A:34;ASN A:34;HIS A:39;CYS A:47;SER A:49;LYS A:137;ASP A:157
Delphinidin 3‐5‐diglucoside	PTGS2	5F19	−9.4	0.75	Hbond	14	ALA A:199;GLN A:203;THR A:206;THR A:206;HIS A:207;PHE A:210;PHE A:210;THR A:212;THR A:212;HIS A:214;HIS A:386;HIS A:388;GLN A:454;GLN A:454
Rutin	PTGS2	5F19	−9.2	5.63	Hbond	7	PHE A:210;THR A:212;ASN A:222;ASN A:382;ASN A:382;ASN A:382;HIS A:386
Kaempferol	EGFR	1 m17	−8.7	0.82	Hbond	6	ALA A:719;LYS A:721;GLU A:738;THR A:766;ASP A:831;ASP A:831
Isorhamnetin	EGFR	1 m17	−8.9	0.34	Hbond	8	LYS A:721;GLU A:738;GLU A:738;THR A:766;THR A:766;CYS A:773;ASP A:831;ASP A:831
Hyperoside	EGFR	1 m17	−9.0	1.03	Hbond	8	LYS A:721;LYS A:721;GLU A:738;GLU A:738;LEU A:764;THR A:766;MET A:769;GLY A:772
Isoquercitrin	EGFR	1 m17	−9.0	0.84	Hbond	5	LYS A:721;LYS A:721;THR A:766;MET A:769;GLY A:772
Delphinidin 3‐5‐diglucoside	EGFR	1 m17	−9.8	1.05	Hbond	13	ALA A:719;LYS A:721;GLU A:738;GLU A:738;THR A:766;MET A:769;ARG A:817;ASN A:818;THR A:830;ASP A:831;ASP A:831;ASP A:831;ASP A:831
Rutin	EGFR	1 m17	−9.7	1.08	Hbond	7	GLY A:697;LYS A:721;LYS A:721;GLU A:738;THR A:766;GLY A:772;ASP A:831

In the case of AChE, for instance, hyperoside recorded a binding energy of −10.0 kcal/mol and formed 14 hydrogen bonds RMSD 0.69 Å, indicating a robust inhibitory potential. Delphinidin 3‐5‐diglucoside and rutin likewise displayed very negative docking scores less than −10 kcal/mol and multiple hydrogen bonds, suggesting strong affinities at key residues ASP A:74, TYR A:124, and ARG A:296. For amylase, rutin achieved a binding energy of −9.4 kcal/mol, forming 11 hydrogen bonds; isoquercitrin −8.4 kcal/mol similarly exhibited a stable docking pose RMSD 1.09 Å with a high hydrogen bond count. With regard to BChE, isoquercitrin and rutin each demonstrated binding energies around −10.3 to −10.8 kcal/mol, forming over 10 hydrogen bonds apiece, thereby suggesting notable inhibitory activity (Dubey et al. 2017; Szwajgier et al. 2020). Analysis of EGFR highlighted delphinidin 3‐5‐diglucoside −9.8 kcal/mol; 13 hydrogen bonds and rutin −9.7 kcal/mol; 7 hydrogen bonds as potent binders (Figure 4D). Evaluations of GTPase KRas confirmed that most ligands fell within the −9 to −8 kcal/mol range, although delphinidin 3‐5‐diglucoside reached 15 hydrogen bonds RMSD 0.83 Å, suggesting significant stabilization within the binding pocket (Figure 4B). For glucosidase, isoquercitrin −8.0 kcal/mol and rutin −9.2 kcal/mol both satisfied the cutoffs for RMSD less than or equal to two angstroms and hydrogen bond count greater than or equal to four, indicating moderate to good inhibitory prospects. Meanwhile, IGFR1 recognized hyperoside, delphinidin 3‐5‐diglucoside, and rutin as plausible ligands, with binding energies clustered around −8.4 to −9.1 kcal/mol. Assessment of PTGS2 also underscored delphinidin 3‐5‐diglucoside −9.4 kcal/mol; 14 hydrogen bonds for its relatively strong binding (Figure 4C). Lastly, for Tyr, only rutin −9.0 kcal/mol; 12 hydrogen bonds fulfilled the RMSD and hydrogen bond criteria, pointing to a potentially moderate inhibitory affinity (Si et al. 2012). Notably, certain residues emerged as interaction “hotspots” across targets—such as ASP A:74 and ARG A:296 in AChE or GLN A:63 and HIS A:299 in amylase—suggesting recurring binding motifs that may be exploited for further lead optimization. Overall, these findings indicate that low RMSD, higher hydrogen bond counts, and sufficiently negative binding energies less than −9 kcal/mol serve as clear hallmarks of promising inhibitory potential, aligning well with prior models of protein‐ligand stability(Morris et al. 2009; Oleg Trott & Arthur J Olson, 2010). Future research might focus on in vitro validation or structure‐guided modifications to bolster these flavonoids' anticancer efficacy.

#### 2.16.1 Molecular Dynamics Simulation

With an emphasis on clarifying their binding sites, the current study aims to find possible therapeutic drugs by thoroughly examining the molecular interactions between particular ligands and target proteins. The results of the KRas, PTGS2, and EGFR complex with delphinidin 3,5‐diglucoside as complexes were used to select three complexes for evaluation. These complexes exhibited robust selectivity and stability in their interactions, as well as important criteria such as the presence of hydrogen‐bonding residues, binding energy, and RMSD. Subsequently, the complexes underwent molecular dynamics (MD) simulations, which afforded greater insight into their biological efficacy and protein binding capacities, thus facilitating a more comprehensive evaluation of their potential as therapeutic agents.

**FIGURE 5:**
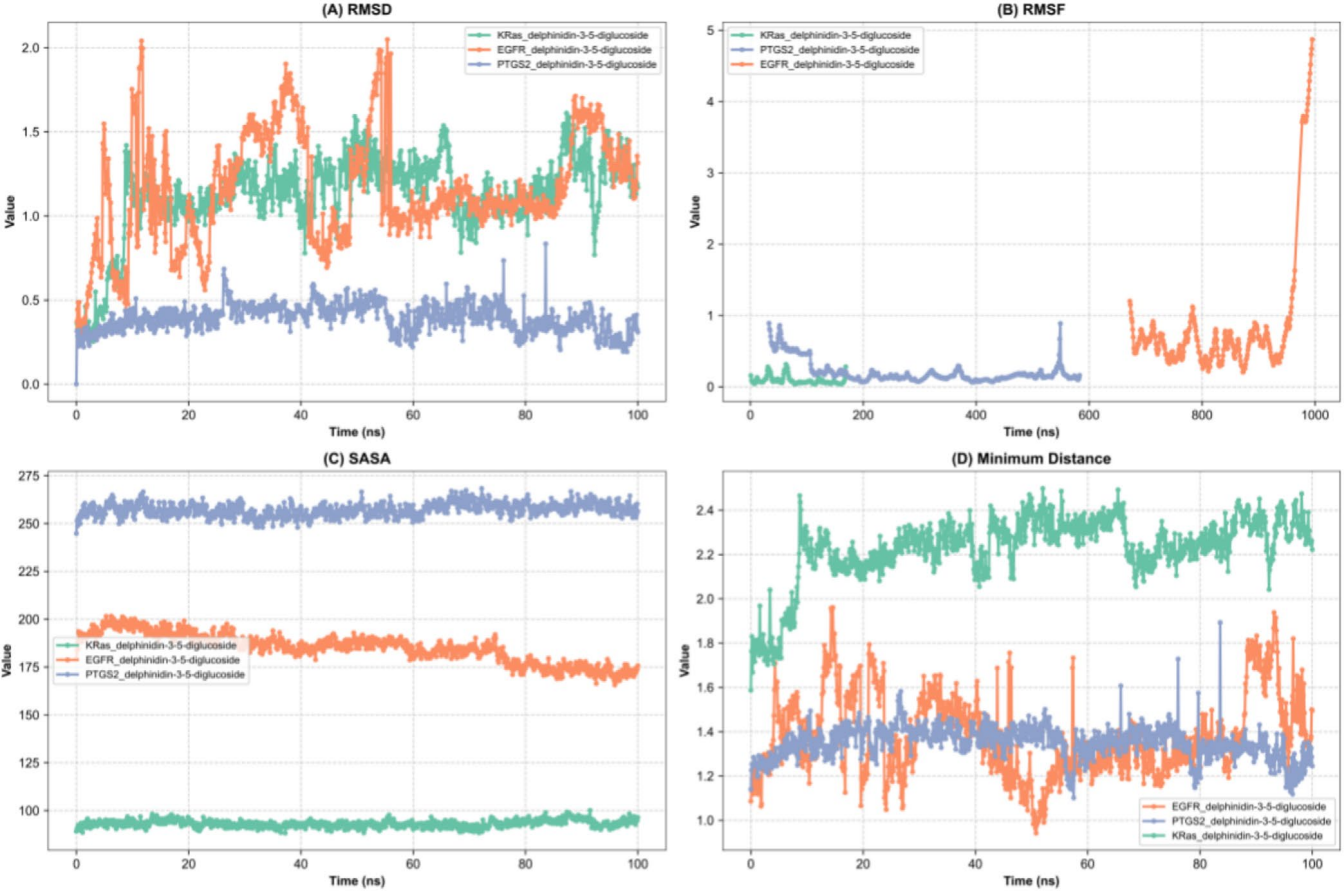
Presentation of molecular dynamics simulations in graphical form; (A) RMSD of GTPase KRas, PTGS2, and EGFR complex with delphinidin 3,5‐diglucoside. (B) RMSF of GTPase KRas, PTGS2, and EGFR complex with delphinidin 3,5‐diglucoside. (C) Solvent Accessibility of GTPase KRas, PTGS2, and EGFR complex with delphinidin 3,5‐diglucoside. (D) Minimum distance of GTPase KRas, PTGS2, and EGFR complex with delphinidin 3,5‐diglucoside.

**FIGURE 6:**
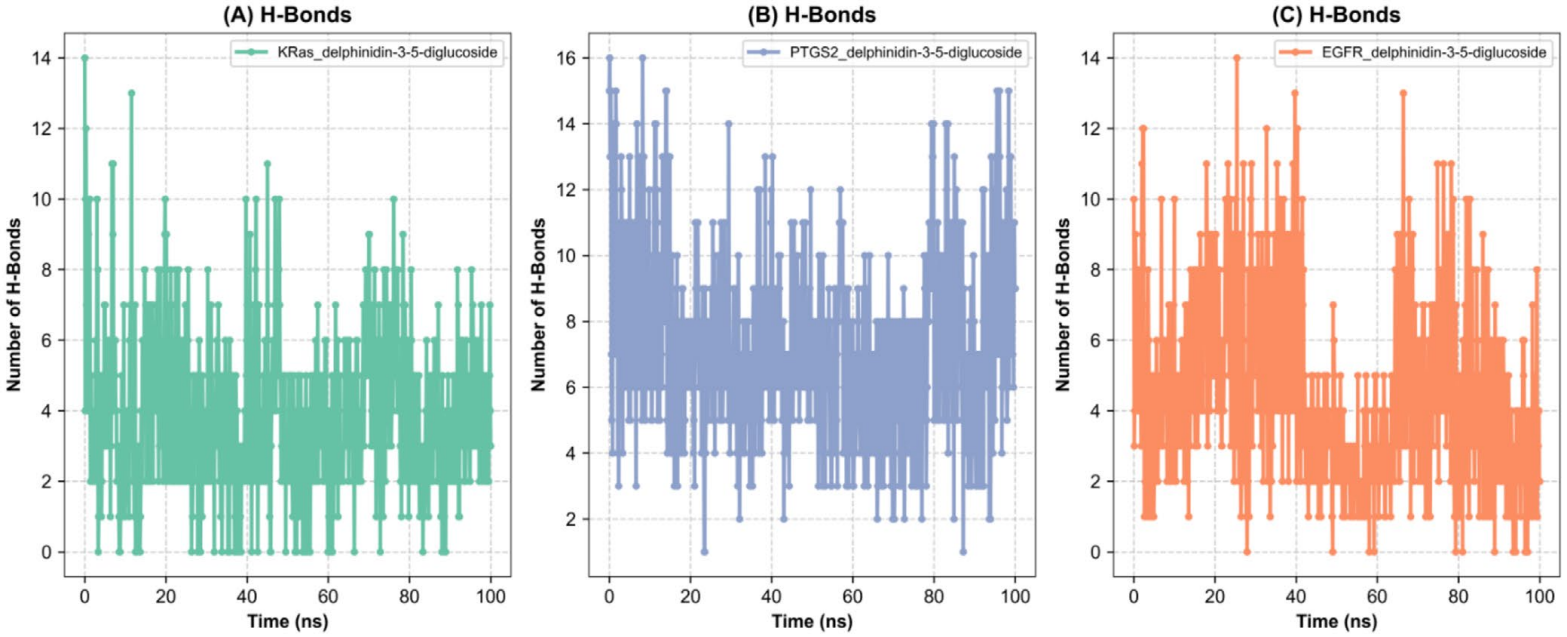
Hydrogen bonds of GTPase KRas, PTGS2, and EGFR complex with delphinidin 3,5‐diglucoside. (A) Hydrogen bonds of GTPase KRas_delphinidin 3,5‐diglucoside complex. (B) Hydrogen bonds of PTGS2_delphinidin 3,5‐diglucoside complex. (C) Hydrogen bonds of EGFR_delphinidin 3,5‐diglucoside complex.

This study demonstrates a correlation between the structural changes and the RMSD of ligand protein complexes, as determined through MD simulations over 100 ns, and the previously conducted analysis. The EGFR_delphinidin‐3‐5‐diglucoside complex exhibited moderate structural fluctuations, with RMSD values ranging from 0 to 2 nm. A gradual increase in RMSD values was observed, reaching a maximum level of broadening at 100 ns. Moderate flexibility alterations were observed. In comparison to the aforementioned structures, the KRas_delphinidin‐3‐5‐diglucoside complex exhibited enhanced stability with RMSD values ranging from 0 to 1.5 nm. The RMSD values for these computations demonstrated consistent stability throughout the majority of the computation, exhibiting minimal fluctuations due to system changes, with occasional brief periods of variation. The PTGS2_delphinidin‐3‐5‐diglucoside complex exhibited overall structural stability, with RMSD values ranging from 0 to 0.5 nm. Although the structure remained largely stable throughout the simulation, occasional minor fluctuations were observed, reflecting transient structural adjustments (Figures 5 and 6A). The present study investigates the flexibility of protein complexes through the analysis of RMSF values obtained from molecular dynamics (MD) simulations. The most prominent peak of the RMSF profile of the PTGS2_delphinidin‐3‐5‐diglucoside complex reaches a value of 0.8906 nm at residue 33. Residues 30–33 are identified as dynamic regions with RMSF > 0.5 nm. In contrast, the KRas_delphinidin‐3‐5‐diglucoside complex demonstrates a maximum RMSF value of 0.3167 nm at residue 63, which is among the lower values observed in most complexes. The region of the EGFR_delphinidin‐3‐5‐diglucoside complex that demonstrated the most flexibility, with a maximum value of 4.8703 nm, was observed in residue 995. Residues 990–995 show RMSF > 1.0 nm, indicating limited dynamic motion. The low RMSF values of PTGS2 and KRas indicate rigid structures, while EGFR shows greater dynamic motion. These findings provide valuable insights into the structural stability and potential biological interactions of the complexes (Figure 6B). The minimum distance in the binding site has been demonstrated to provide critical insights into the structural stability and flexibility of ligand–protein complexes. The PTGS2_delphinidin‐3‐5‐diglucoside complex exhibited distances ranging from 1.10 to 1.89 nm, with an average of 1.35 nm (±0.24 nm). Significant fluctuations were observed, indicating a flexible binding region characterized by periods of compaction and expansion. A similar observation was made in the KRas_delphinidin‐3‐5‐diglucoside complex, which exhibited distances ranging from 1.59 to 2.50 nm, with a mean of 2.23 nm (±0.33 nm), reflecting moderate flexibility and structural stability. In contrast, the EGFR_delphinidin‐3‐5‐diglucoside complex displayed pronounced dynamic behavior, suggesting a highly adaptable binding region with considerable flexibility. These findings underscore substantial disparities in stability and flexibility among the complexes, with PTGS2 and EGFR exhibiting greater adaptability compared to KRas (Figure 6C). The analysis of the solvent accessible surface area (SASA) of the ligand protein complexes suggests the existence of notable differences in the structural dynamics and solvent exposure. On average, the PTGS2_delphinidin‐3‐5‐diglucoside complex has a SASA value of approximately 257.27 nm^2^ with moderate fluctuations of about 23.49 nm^2^. The KRas_delphinidin‐3‐5‐diglucoside complex exhibited average SASA values of 93.13 nm^2^, suggesting a more compact structure with restricted solvent interactions and minimal fluctuations of approximately 11.88 nm^2^. In contrast, the EGFR_delphinidin‐3‐5‐diglucoside complex exhibited a higher mean value of solvent accessible surface area (SASA) at 185.10 nm^2^, along with substantial variations of approximately 35.90 nm^2^. This observation is indicative of an open conformation and an enhancement in dynamic structural flexibility. Hydrogen bond analysis is a method that provides insights into the stability and dynamics of ligand–protein complexes. The PTGS2_delphinidin‐3‐5‐diglucoside complex demonstrated hydrogen bond fluctuations between 1 and 16 throughout the simulation, with an average of 6 during the intermediate stage (20–80 ns) and recovering to 8 after 80 ns, suggesting conformational adjustments and regained stability. In contrast, the KRas_delphinidin‐3‐5‐diglucoside complex exhibited a reduced number of hydrogen bonds (0–14), with averages dropping from 4 during the initial stage (0–20 ns) to 3 during the intermediate and final stages. This finding suggests a decline in stability and an increase in flexibility over time (Figure 6A). Conversely, the EGFR_delphinidin‐3‐5‐diglucoside complex demonstrated a more extensive array of hydrogen bonds (0–14), with averages decreasing from 5 during the initial stage to 4 after 80 ns, suggesting substantial reductions in stability and a highly dynamic binding region (Figure 6C).

## 3 Conclusion

The present study demonstrated for the first time the phenolic profile, antioxidant, enzyme inhibition, and cytotoxic properties of *B. trixago*. Results revealed that the tested extracts varied in their phytoconstituent content and biological activities. Generally, most of the identified compounds were detected in all extracts, but with variable concentrations. EtOH extract recovered the highest TPC and TFC and the highest content of most of the identified compounds. Rutin comprised the major constituent of the EtOH and 70% EtOH extracts, while 4‐hydroxybenzoic represented the major compound in the aqueous and EtOAc extracts. The 70% EtOH and aqueous extracts displayed higher antioxidant activity in most assays (4/6) while the EtOH and/or EtOAc extracts exerted the best cholinesterase, α‐amylase, and α‐glucosidase inhibition activity. All extracts except the aqueous showed comparable antityrosinase activity. The plant was most cytotoxic toward the HT‐29 cell line, where the best effect was exerted, respectively, by the 70% EtOH and aqueous extracts. In additional analyses, network pharmacology, molecular docking, and molecular dynamics simulations demonstrated the therapeutic relevance of the bioactive compounds, particularly in colon adenocarcinoma. The results revealed significant protein–ligand interactions and pathway modulations, supporting their potential role in colon adenocarcinoma‐related mechanisms as well as their possible implications in neurodegenerative, metabolic, and skin disorders. Thus, *B. trixago* could be a promising source of bioactive molecules with interesting pharmacological applications. Our future study will focus on the isolation and identification of more compounds in addition to evaluating their therapeutic potential.

## Author Contributions

**Mehmet Veysi Cetiz:** conceptualization (equal), data curation (equal), visualization (equal), writing – original draft (equal), writing – review and editing (equal). **Sakina Yagi:** data curation (equal), investigation (equal), methodology (equal), writing – original draft (equal), writing – review and editing (equal). **Kouadio Ibrahime Sinan:** investigation (equal), methodology (equal), validation (equal), visualization (equal), writing – original draft (equal). **Ismail Senkardes:** investigation (equal), methodology (equal), resources (equal), writing – original draft (equal). **Ismail Koyuncu:** data curation (equal), investigation (equal), methodology (equal), supervision (equal), writing – original draft (equal), writing – review and editing (equal). **Ozgur Yuksekdag:** investigation (equal), methodology (equal), visualization (equal), writing – original draft (equal). **Giovanni Caprioli:** data curation (equal), investigation (equal), methodology (equal), writing – original draft (equal), writing – review and editing (equal). **Agnese Santanatoglia:** data curation (equal), investigation (equal), methodology (equal), writing – original draft (equal). **Gianni Sagratini:** data curation (equal), investigation (equal), methodology (equal), writing – original draft (equal). **Enver Saka:** investigation (equal), methodology (equal), validation (equal), writing – original draft (equal). **Gulsah Ozturk:** investigation (equal), methodology (equal), software (equal), writing – original draft (equal). **Bengusu Hacer Akgul:** investigation (equal), methodology (equal), writing – original draft (equal), writing – review and editing (equal). **Gokhan Zengin:** conceptualization (equal), data curation (equal), funding acquisition (equal), investigation (equal), methodology (equal), supervision (equal), writing – original draft (equal), writing – review and editing (equal).

## Conflicts of Interest

The authors declare no conflicts of interest.

## Data Availability

Data will be made available on request.
